# The relationship between physical activity levels and serum vitamin D levels varies among children and adolescents in different age groups

**DOI:** 10.3389/fnut.2024.1435396

**Published:** 2024-08-30

**Authors:** Shengrong Ouyang, Qin Li, Zhuo Liu, Yan Yin

**Affiliations:** ^1^Department of Biochemistry and Immunology, Capital Institute of Pediatrics, Beijing, China; ^2^Environmental Standards Institute, Chinese Research Academy of Environmental Sciences, Beijing, China; ^3^Department of Integrated Early Childhood Development, Capital Institute of Pediatrics, Beijing, China

**Keywords:** physical activity, vitamin D, children, adolescents, different ages

## Abstract

**Objective:**

The objective of the present study was to explore the relationship between physical activity (PA) levels and serum vitamin D levels in children and adolescents of different ages and sexes.

**Methods:**

All the data in this study were collected during two cycles (2011–2014) of the National Health and Nutrition Examination Survey (NHANES). Our study participants were aged ≥3 and < 20 years and had valid data for all variables, including vitamin D intake, serum vitamin D levels, PA volume and intensity levels, amount of time spent outdoors, body mass index (BMI), sex, and race.

**Results:**

A total of 3,312 participants were included in the study; 1,672 were boys (50.4%), and 1,640 were girls (49.6%). A total of 250 (7.5%) children were aged 3–5 years, 1,474 (44.5%) were aged 6–11 years, and 1,588 (47.9%) were aged 12–19 years. Both PA volume and intensity were positively related to serum vitamin D levels in the 6–11-year-old boys and girls (*p* < 0.05 for both) and in the 12–19-year-old boys. No significant relationship between PA volume or intensity and serum vitamin D levels was detected in the 3–5-year-old group or in the 12–19-year-old girl group. The time spent outdoors and the BMI of the participants had mediating effects on the relationships of PA volume and intensity with serum vitamin D levels in boys and girls aged 6–11 years.

**Conclusion:**

The relationship between PA and vitamin D varies among children and adolescents of different sexes and ages, and the sun exposure level and BMI had mediating effects on the relationship between PA and the serum vitamin D level. The mechanism of the relationship between PA and increased serum vitamin D levels needs further in-depth research.

## Introduction

Vitamin D controls plasma calcium levels after intestinal absorption, helping to regulate bone metabolism (1). In addition to calcium metabolism, vitamin D plays an important role in exoskeleton tissues such as pancreatic tissue, adipocytes, and skeletal muscle and is involved in regulating immune responses (2). Due to rapid skeletal growth and the development of many organ systems during childhood and adolescence, vitamin D levels may be particularly important during this period.

However, vitamin D deficiency has been reported worldwide. Akkermans et al. (3) studied 325 children in Western Europe and reported that the overall prevalence of vitamin D deficiency was 22.8%. The prevalence of vitamin D insufficiency in Iranian children and adolescents was 31% (4). A multicenter, hospital-based, cross-sectional observational study from China surveyed 465,337 children from 825 hospitals in 18 provinces and reported that the prevalence rates of vitamin D deficiency (<30 nmol/L) and insufficiency (30–50 nmol/L) were 6.69 and 15.92%, respectively (5).

Vitamin D can be obtained from exposure to UV radiation from the sun, the diet, or supplements. The main source of vitamin D for humans is exposure of the skin to ultraviolet B (UVB) radiation (290–315 nm) from the sun. (6) Skin synthesis is estimated to provide 80–100% of the body’s vitamin D requirements (7). However, excessive sun exposure can increase the risk of skin aging and skin cancer (8); moreover, several factors that hinder year-round synthesis, such as season, latitude, major weather conditions (9), and sunscreen use (9), have been identified.

Diets contain relatively low levels of vitamin D. Many studies have shown that vitamin D supplements and vitamin D-fortified foods significantly improve vitamin D status (10). However, only consumers obtain the benefits of supplements (11, 12). Moreover, incorrect supplementation methods, such as intermittent and high-dose treatment, may lead to unexpected adverse reactions (10).

In recent years, many studies have shown that physical activity (PA) is associated with an increase in adult vitamin D levels, excluding the impact of sunlight exposure on vitamin D levels (13, 14). Although the ability of sunlight to synthesize vitamin D decreases with age, studies targeting elderly people have shown the same results (13). Several studies have also shown a positive correlation between vitamin D levels and PA levels in children and adolescents (15, 16).

Physical activity is any movement caused by muscle contraction, which leads to an increase in energy expenditure compared to that at rest (17). PA has a positive impact on health (18). Research has confirmed that in the National Health and Nutrition Examination Survey (NHANES) population, individuals who engage in PA have a lower mortality rate (18). In the adult population, a large amount of research evidence supports the positive role of exercise interventions in improving human metabolic parameters, including lipid status, insulin resistance markers, and the levels of other related hormones (219). Research on the child population has also confirmed the role of exercise training in reducing insulin resistance (19, 20) and improving cardiac metabolic health (21, 22). Therefore, the use of PA to improve vitamin D status will provide additional and profound benefits.

However, is the relationship between PA and vitamin D affected by the physiological differences between boys and girls, the varying physical abilities at different ages, and the many physical changes that occur during adolescence? What is the relationship between PA and vitamin D levels in children of different ages and sexes? Answering these questions will provide more scientific and specific guidance for children and adolescents to adopt measures to improve vitamin D status through PA. To our knowledge, little research has been conducted on the relationship between PA and vitamin D levels in individuals of different ages and sexes. Therefore, studies exploring the relationship between PA and vitamin D levels in children and adolescents of different sexes and ages and those recommending an appropriate mode to increase vitamin D levels are necessary.

Therefore, we used data from the NHANES, which uses precise motion accelerometers to record the amount and intensity of PA, to explore the effects of PA on serum vitamin D levels in preschoolers, schoolchildren, and adolescents, which can provide a scientific basis for the study of PA in improving children’s vitamin D levels according to sex and age.

## Materials and methods

### Study population

In this study, all data were derived from the NHANES, a nationwide assessment that evaluates the health and nutritional status of both adults and children in the United States. The NHANES uses a stratified, multistage random sampling approach to ensure representative sampling. The survey data are updated and made publicly accessible every 2 years. For this research, we analyzed data from two consecutive NHANES cycles spanning the years 2011 to −2014. Our study participants were children and adolescents aged ≥3 and < 20 years who had valid data for serum vitamin D and PA levels. The NHANES protocol was approved by the National Center for Health Statistics Research Ethics Review Board (Protocol #2011-17).

### Variables

#### Physical activity

In 2011–2014, the NHANES utilized ActiGraph GT3X+ wrist-worn accelerometers (Pensacola, FL, United States) to assess physical activity. These devices captured triaxial acceleration data along the *x*-, *y*-, and *z*-axes. The data were translated into Monitor-Independent Movement Summary (MIMS) units on a minute-by-minute basis, employing a universal, device-agnostic algorithm. This approach facilitated consistent comparisons across diverse studies and research designs (23). The data collected on the first and last partial days were excluded from the analysis before any of the MIMS metrics were calculated (24). We excluded “nonwear” and “unknown” minutes, including only “wear” (“wake” and “sleep”) minutes. Participants who wore the device for more than 10 wear hours (“wake” and “sleep”) per day, who had no less than 3 “sleep” wear hours and 7 “wake” wear hours, respectively, and who wore it for at least 3 valid days were included (24).

In the present study, the MIMS metrics encompassed both PA volume, represented as the average daily MIMS units, reflecting the total number of MIMS units accumulated daily across valid assessment days, and PA intensity, which is quantified by the peak 60-min MIMS value (23). The peak 60-min MIMS value was defined as the average movement per day, encompassing the 60 highest MIMS units per minute (not necessarily consecutive) across all valid assessment days. This metric was calculated by first ranking an individual’s MIMS units per minute for each valid day, determining the mean of the top 60 values within each day, and finally averaging these per-minute MIMS units across all valid wear days (24). The use of the peak 60-min MIMS value is consistent with the daily guidelines for moderate-to-vigorous aerobic PA among children and adolescents (25) and partly mirrors the peak 60-min stepping cadence employed in previous studies (23).

#### Vitamin D

The serum vitamin D level (nmol/L) was determined in this study by summing the 25-hydroxyvitamin D2 and 25-hydroxyvitamin D3 levels. Ultrahigh-performance liquid chromatography–tandem mass spectrometry was utilized for the quantitative detection of vitamin D levels. The laboratory procedure manual outlines the methodologies adopted for collecting, transporting, storing, and analyzing vitamin D samples (26, 27).

#### Covariates

The demographic variables included age, sex, race (categorized as Mexican American, other Hispanic, non-Hispanic white, non-Hispanic black, and other), and the family poverty–income ratio (PIR). Additionally, body mass index (BMI) was computed by dividing an individual’s weight in kilograms by the square of their height in meters. The weight status of children under 20 years of age was evaluated using the 2000 Centers for Disease Control and Prevention (CDC) growth charts, where obesity was defined as a BMI equal to or surpassing the 95th percentile of the sex-specific BMI-for-age percentiles, overweight encompassed a BMI ranging from the 85–95th percentiles, and normal weight corresponded to a BMI falling between the 5th and 85th percentiles (28)Furthermore, the total intake of vitamin D and the amount of time spent outdoors are considered two potential factors influencing serum vitamin D levels. Total vitamin D intake is derived from daily food and vitamin supplements. Individuals who consumed more than 100 μg of vitamin D per day were excluded. The amount of time spent outdoors (outdoor time) was measured through ambient light levels recorded by ActiGraph model GT3X+ accelerometers. The time spent in outdoor and indoor locations was determined by considering lux values, where ≥240 lux indicates outdoor locations and < 240 lux indicates indoor locations (27). Notably, a previous study demonstrated that a threshold of 240 lux achieved a remarkable 97% accuracy in distinguishing between indoor and outdoor conditions in a naturalistic setting (27). The daily outdoor time for each individual, reported in minutes per day, was calculated by averaging the outdoor minutes across valid wear days. Three participants were excluded because they spent more than 7 h outdoors per day, which was more than 4 times the mean daily amount of time spent outdoors.

### Statistical analysis

Aggregate statistics were generated for the outcomes, exposures, and relevant covariates. Normally distributed continuous variables are presented as the means ± SDs, whereas nonnormally distributed continuous variables are presented by as medians (IQRs). First, we employed the Kruskal–Wallis rank sum test and the chi-square test to assess the subgroup differences according to sex (male and female) and age group (3–5, 6–11, and 12–19 years) in the descriptive analysis of the baseline characteristics of the participants. Stratified multivariate linear regressions were performed to analyze the associations between the serum vitamin D level and the PA volume or PA intensity for different age and sex groups. The serum vitamin D level was the dependent variable. Sun exposure is the main source of vitamin D in the human body. Two adjusted models were used in the analyses to better illustrate the role of sun exposure in the relationship between PA and serum vitamin D levels. Model 1 was adjusted for race/ethnicity, BMI category, PIR, and total vitamin D intake. Model 2 further incorporated the amount of time spent outdoors time in addition to all the variables in Model 1.

After identifying subgroups in which a significant correlation existed between PA and the serum vitamin D level through a stratified multivariate linear regression analysis across the two models, we proceeded to evaluate the potential mediating effects of BMI and the amount of time spent outdoors on the relationship between PA and the serum vitamin D level within these identified subgroups using a mediation model.

These mediation models were adjusted for race/ethnicity, BMI, PIR, total vitamin D intake, and the amount of time spent outdoors, except when covariates such as BMI or outdoor time were used as mediating variables. The average direct effect represents the effects of PA levels on serum vitamin D levels without a mediator. The average causal mediation effect (ACME) indicates that the influence of PA levels on serum vitamin D levels is mediated through BMI or outdoor time as an intermediary factor. The proportion of mediation was determined by dividing the ACME by the total effect. The proportion mediated was estimated when the mediated effect was significant.

Statistical analyses were performed using R software 4.2.0 (The R Foundation, http://www.R-project.org). The mediation analyses were executed via the “mediation” R package (version 4.5.0). All the statistical tests were conducted with two-sided significance, and a *p* value less than 0.05 was considered to indicate statistical significance.

## Results

### Study participants and baseline characteristics

**Figure 1:**
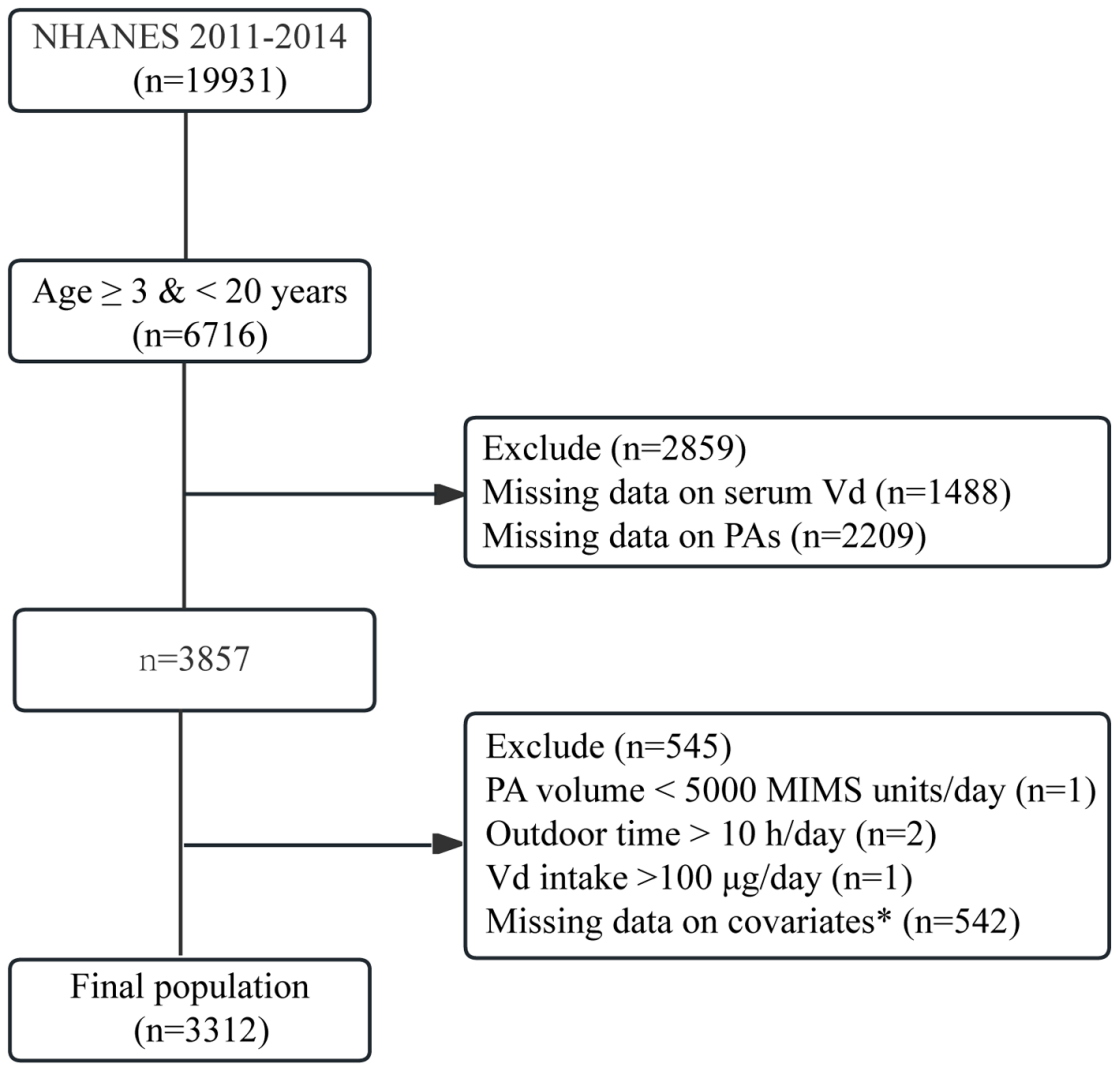
Flowchart of participant selection. ^*^Covariates included age, sex, race/ethnicity, body mass index, poverty–income ratio, and Vd intake.

Among the 19,931 original cohort members, 3,857 individuals were aged 3–20 years and had no missing information on vitamin D levels, PA levels or the amount of time spent outdoors. Among these 3,854 individuals, three were excluded for excessive vitamin D intake or excessive time spent outdoors. Additionally, 542 individuals with missing information on the covariates were further excluded. Ultimately, 3,312 eligible participants were included in this study. The selection flow chart is shown in Figure 1.

A total of 3,312 participants were included in the study; 1,672 were boys (50.4%), and 1,640 were girls (49.6%). A total of 250 (7.5%) children were aged 3–5 years, 1,474 (44.5%) were aged 6–11 years, and 1,588 (47.9%) were aged 12–19 years. As shown in Table 1, no significant differences in the sex distributions of the 3–5-year-old group, the 6–11-year-old group, or the 12–19-year-old group were observed (*p* > 0.05).

**Table 1:** Study participants and baseline characteristics.

	3–5-year-old group			6–11-year-old group			12–19-year-old group		
Characteristic	Male	Female	*p*	Male	Female	*p*	Male	Female	*p*
*N* (%)	126 (50%)	124 (50%)		749 (51%)	725 (49%)		797 (50%)	791 (50%)	
Race/ethnicity^#^			>0.9			0.3			0.088
*Mexican American*	35 (28%)	32 (26%)		159 (21%)	175 (24%)		157 (20%)	173 (22%)	
*Other Hispanic*	15 (12%)	13 (10%)		83 (11%)	63 (8.7%)		75 (9.4%)	91 (12%)	
*Non-Hispanic White*	25 (20%)	30 (24%)		214 (29%)	185 (26%)		220 (28%)	185 (23%)	
*Non-Hispanic Black*	27 (21%)	27 (22%)		201 (27%)	206 (28%)		225 (28%)	202 (26%)	
*Other/Multiracial*	24 (19%)	22 (18%)		92 (12%)	96 (13%)		120 (15%)	140 (18%)	
BMI category^#^			0.3			0.3			0.7
*Underweight*	4 (3.2%)	10 (8.1%)		22 (2.9%)	12 (1.7%)		25 (3.1%)	21 (2.7%)	
*Normal weight*	92 (73%)	83 (67%)		416 (56%)	403 (56%)		447 (56%)	462 (58%)	
*Overweight*	16 (13%)	13 (10%)		128 (17%)	140 (19%)		137 (17%)	137 (17%)	
*Obese*	14 (11%)	18 (15%)		183 (24%)	170 (23%)		188 (24%)	171 (22%)	
PIR^*^	1.89 ± 1.48	1.89 ± 1.59	0.6	1.91 ± 1.52	1.87 ± 1.51	0.6	2.11 ± 1.59	1.96 ± 1.53	<0.05
Total vitamin D intake^*^ (μg)	6.3 (3.9, 12.9)	7.0 (3.6, 12.2)	0.9	6.7 (4.4, 10.7)	6.0 (3.6, 9.8)	<0.05	5.4 (2.7, 10.0)	3.8 (2.0, 6.7)	<0.05
Serum vitamin D^*^ (nmol/L)	72.4 ± 16.6	69.4 ± 17.6	0.2	65.8 ± 16.5	62.1 ± 18.9	<0.05	57.4 ± 18.7	54.8 ± 21.8	<0.05
PA volume^*^ (MIMS Units/day)	19,813 ± 2,859	19,127 ± 2,850	0.080	19,435 ± 3,530	19,020 ± 3,082	<0.05	14,270 ± 3,604	14,419 ± 3,189	<0.05
PA intensity^*^ (MIMS units/min)	64.2 ± 10.7	58.9 ± 9.3	<0.05	65.9 ± 12.3	59.7 ± 10.2	<0.05	49.3 ± 11.6	45.8 ± 7.6	<0.05
Outdoor time^*^ (min/day)	95.6 ± 60.0	91.4 ± 61.6	0.5	107.6 ± 66.7	99.0 ± 61.1	<0.05	100.8 ± 67.9	81.1 ± 56.9	<0.05

^*^Means ± SDs; medians (IQRs); the Wilcoxon rank sum test was applied. ^#^Pearson’s chi-square test was applied. Outdoor time, the amount of time spent outdoors.

As shown in Table 1, among the participants, 94 children (2.8%), including 51 boys and 43 girls, had a BMI < 5th percentile, and 1,315 (39.7%) had a BMI ≥ 85th percentile. The intake of vitamin D by girls aged 6–11 and 12–19 years was lower than that of boys in the corresponding age groups.

#### Distribution of vitamin D levels

**Figure 2:**
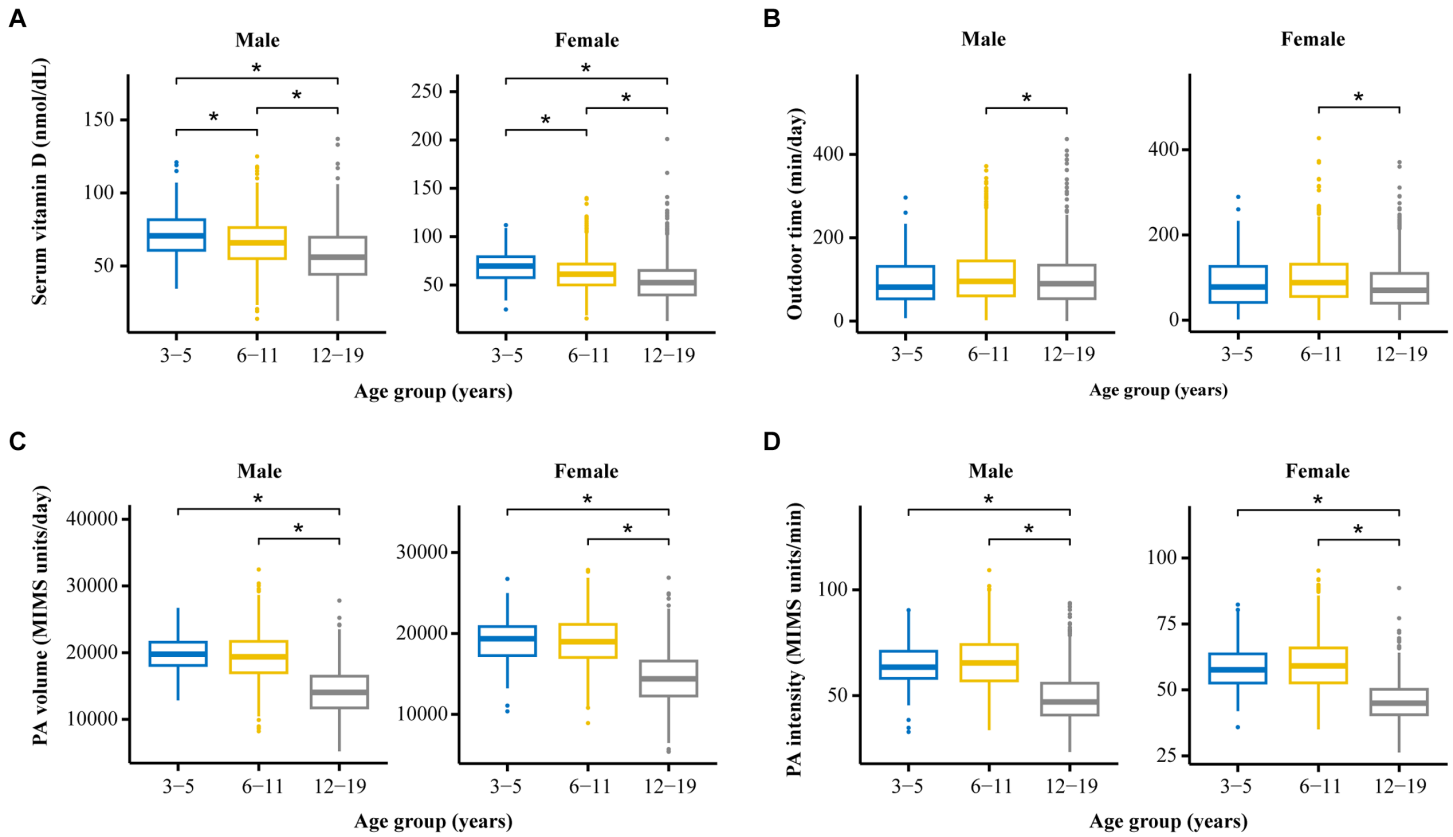
The distribution and differences among four crucial variables (vitamin D level, PA volume, PA intensity, and outdoor time) among groups stratified by age and sex. **(A)** Comparison of serum vitamin D levels among participants of different ages and different sexes. **(B)** Comparison of outdoor time levels among participants of different ages and different sexes. **(C)** Comparison of PA volumes among participants of different ages and different sexes. **(D)** Comparison of PA intensity levels among participants of different ages and different sexes. ^*^p < 0.05.

As shown in Table 1 and Figure 2, for children of the same sex, the serum vitamin D levels in the 12–19-year-old group (males, 57.4 ± 18.7 nmol/L; females, 54.8 ± 21.8 nmol/L) were lower than those in the 6–11-year-old group (males, 65.8 ± 16.5 nmol/L; females, 62.1 ± 18.9 nmol/L), and the 3–5-year-old group (males, 72.4 ± 16.6 nmol/L; females, 69.4 ± 17.6 nmol/L).

The serum vitamin D levels of the girls in the 6–11-year-old group and in the 12–19-year-old group were lower than those of the boys in the corresponding age groups. A statistically significant difference in vitamin D levels was not observed between boys and girls in the 3–5-year-old group.

#### Distribution of PA volume according to age and sex

As shown in Table 1 and Figure 2, for individuals of the same sex, the PA volume in the 12–19-year-old group was lower than that in the 3–5-year-old group and the 6–11-year-old group for boys or girls (*p* < 0.05). No statistically significant difference in the PA volume was observed between the 3–5-year-old group and the 6–11-year-old group of boys or girls.

Within the same age groups, a statistically significant difference in PA volume was not observed between boys and girls in the 3–5-year-old group or the 12–19-year-old group (*p* > 0.05). The PA volume of boys was greater than that of girls in the 6–11-year-old group (*p* < 0.05).

#### Distribution of PA intensity levels in children of different sexes and ages

As shown in Table 1 and Figure 2, for children of the same sex, the PA intensity was significantly lower in the 12–19-year-old group than in the 3–5-year-old group and the 6–11-year-old group (*p* < 0.01) of boys or girls. Moreover, a statistically significant difference in PA intensity was not observed between the 3–5-year-old and the 6–11-year-old groups (*p* > 0.05) of boys or girls.

Similarly, the PA intensity of boys in the 3–5-year-old group, the 6–11-year-old group, and the 12–19-year-old group was greater than that of girls in the corresponding age group (*p* < 0.05).

#### Distribution of the amount of time spent outdoors by children of different sexes and ages

As shown in Table 1 and Figure 2, for children of the same age, the amount of time spent outdoors by girls in the 12–19-year-old group and the 6–11-year-old group was significantly less than that spent by boys in the corresponding group (*p* < 0.05). A statistically significant difference in the amount of time spent outdoors was not observed between males and females in the 3–5-year-old group.

According to the analysis of children of the same sex, girls in the 12–19-year-old group spent less time outdoors than did those in the 3–5-year-old group and the 6–11-year-old group; moreover, no statistically significant difference in this parameter was observed for boys among the three age groups.

### Relationships between PA and serum vitamin D levels in different sex and age groups

#### Relationship between the PA volume and the serum vitamin D level

**Figure 3:**
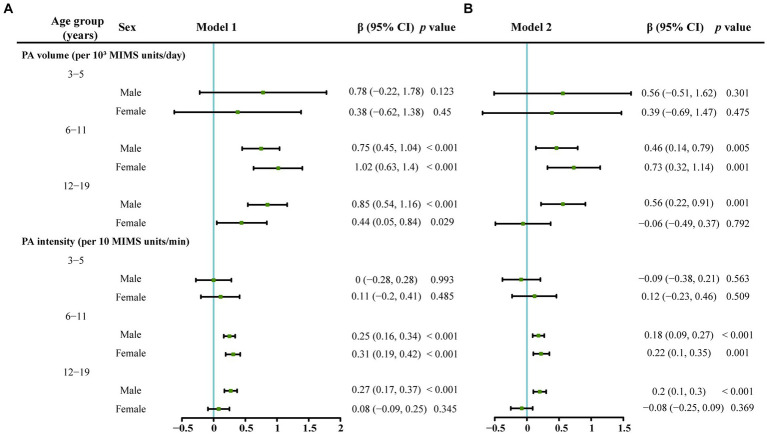
The associations between serum vitamin D levels and PA volume or PA intensity stratified by age and gender analyzed using a multivariate linear regression model. The serum vitamin D level was the dependent variable. **(A)** Model 1 which was adjusted for race/ethnicity, the classification of the body mass index, poverty–income ratio and vitamin D intake. **(B)** Model 2 which was additionally adjusted for outdoor time based on Model 1.

As shown in Figure 3, after excluding the impacts of the amount of time spent outdoors, race/ethnicity, BMI, PIR, and total vitamin D intake, no correlation was detected between the PA volume and serum vitamin D levels in boys in the 3–5-year-old group. A positive correlation was identified between the PA volume and serum vitamin D levels in both boys and girls in the 6–11-year-old group.

In the 12–19-year-old group, a positive correlation was observed between the PA volume and serum vitamin D levels only in boys in Model 1 and Model 2. In the 12–19-year-old female group, a positive correlation was observed between the PA volume and serum vitamin D levels only in Model 1, which was adjusted for race/ethnicity, BMI, PIR, and total vitamin D intake, and no correlation between the PA volume and serum vitamin D levels was found in Model 2, which was adjusted for all the variables in Model 1 plus the amount of time spent outdoors.

#### Relationship between PA intensity and serum vitamin D levels

As shown in Figure 3, in the 3–5-year-old group, PA intensity was not related to the serum vitamin D level (*p* > 0.05). In the 6–11-year-old group, a positive correlation was identified between the PA intensity and serum vitamin D levels in boys and girls in Model 1 and Model 2 (*p* < 0.05). In the 12–19-year-old group, a positive correlation was observed between the PA intensity and serum vitamin D levels only in boys in Model 1 and Model 2 (*p* < 0.05).

### The mediating effects of the amount of time spent outdoors and BMI on the relationship between PA and vitamin D levels

As shown in Table 2, the amount of time spent outdoors had a mediating effect on the relationship between serum vitamin D levels and PA, including the PA volume and PA intensity, in boys aged 12–19 years and in girls and boys aged 6–11 years.

**Table 2:** Estimated proportions of the associations between different dependent variables and serum Vitamin D levels mediated by BMI and the amount of time spent outdoors in the subgroups.^*^

Variable	Sex	Age group (years)	Total effect (95% CI)	Direct effect (95% CI)	Mediated effect (95% CI)	Proportion mediated, %
BMI						
PA volume (per 10^3^ MIMS units/day)						
	Male	6–11	0.53 (0.19, 0.84)	0.35 (0.02, 0.68)	0.18 (0.09, 0.27)	33
	Female	6–11	0.77 (0.36, 1.16)	0.61 (0.2, 1)	0.16 (0.08, 0.25)	21
	Male	12–19	0.57 (0.22, 0.9)	0.52 (0.15, 0.85)	0.05 (−0.02, 0.12)	–
PA intensity (per 10 MIMS units/min)						
	Male	6–11	0.21 (0.13, 0.29)	0.15 (0.06, 0.23)	0.06 (0.03, 0.09)	30
	Female	6–11	0.25 (0.14, 0.37)	0.17 (0.04, 0.28)	0.09 (0.05, 0.13)	34
	Male	12–19	0.23 (0.13, 0.33)	0.18 (0.08, 0.28)	0.05 (0.03, 0.08)	22
Outdoor time						
PA volume (per 10^3^ MIMS units/day)						
	Male	6–11	0.65 (0.35, 0.94)	0.35 (0.02, 0.68)	0.3 (0.15, 0.45)	46
	Female	6–11	0.91 (0.53, 1.28)	0.61 (0.2, 1)	0.3 (0.15, 0.49)	33
	Male	12–19	0.82 (0.47, 1.14)	0.52 (0.15, 0.85)	0.3 (0.13, 0.48)	36
PA intensity (per 10 MIMS units/min)						
	Male	6–11	0.22 (0.13, 0.3)	0.15 (0.06, 0.23)	0.07 (0.04, 0.11)	33
	Female	6–11	0.26 (0.13, 0.37)	0.17 (0.04, 0.28)	0.09 (0.05, 0.15)	36
	Male	12–19	0.25 (0.15, 0.35)	0.18 (0.08, 0.28)	0.07 (0.03, 0.11)	28

^*^The model was adjusted for race/ethnicity, BMI, poverty–income ratio, Vitamin D intake and the amount of time spent outdoors, except when the variable was removed due to its role as a mediating variable.

As shown in Table 2, the smallest mediating effect of the amount of time spent outdoors on the relationship between PA and serum vitamin D levels was 28%, and the largest mediating effect of the amount of time spent outdoors on the relationship between PA and serum vitamin D levels was 46%.

Body mass index had a mediating effect on the relationship between serum vitamin D levels and PA, including the PA volume and PA intensity, whereas BMI had no mediating effect on the relationship between the PA volume and serum vitamin D levels only in boys aged 12–19 years. As shown in Table 2, the range of the proportion of the mediating effects of BMI on the relationship between PA and vitamin D levels was 21–34%.

## Discussion

The present study showed that the relationship between PA and vitamin D varies among different age and sex groups. We found no correlation between PA, including the PA volume and PA intensity, and serum vitamin D levels in boys and girls aged 3–5 years, but we did observe a positive correlation between the PA volume or PA intensity and vitamin D levels in boys and girls aged 6–11 years and in boys aged 12–19 years. These findings are similar to those of previous studies.

Kyungchul Song et al. studied 3,183 participants aged 12–18 years in the Korea National Health and Nutrition Examination Survey (KNHANES) and reported that individuals with normal vitamin D levels had greater PA levels than individuals with vitamin D deficiency (15). Al Othman et al. (16) conducted a cross-sectional study among 331 children aged 6–17 years (153 boys and 178 girls) in Saudi Arabia and reported that, for an equivalent duration of sunlight exposure, individuals with moderate-to-high physical activity levels presented higher levels of vitamin D. Kim et al. (29) used data from the KNHANES to study the relationship between PA levels and vitamin D levels in adolescents and reported that those who did not participate or who only participated in PA for 1–3 days presented a greater prevalence of vitamin D deficiency than did those who engaged in 4–7 days of activity per week. Our research results are similar to those of the studies described above; however, no relationship was observed between PA and serum vitamin D levels in the groups of girls aged 12–19 years.

Van den Heuvel et al. (30) evaluated the effects of PA characteristics (such as duration and intensity) on plasma vitamin D levels and reported that high-intensity PA is positively correlated with vitamin D levels. A lower level of PA intensity may be one of the reasons that PA was related to the serum vitamin D level in girls and boys aged 12–19 years in our study.

Our research showed that for children aged 3–5 years, regardless of sex, no relationship existed between the PA duration or intensity and serum vitamin D levels. Similarly, Charlotte Mortensen’s (31) study revealed a close correlation between vitamin D levels in 4–8-year-old children and sunlight exposure, whereas PA levels in 4–8-year-old children were not related to vitamin D levels. Moreover, our research revealed no statistically significant differences in the duration or intensity of PA between children aged 3–5 years and those aged 6–11 years. Why is no correlation observed between PA and vitamin D levels in this age group? We did not find any further research on the relationship between PA and vitamin D levels in children aged 3–5 years.

We found a correlation between PA and vitamin D levels in children aged 6–11 years. However, some studies have shown that athletes have a greater prevalence of vitamin D deficiency, which is a very prominent problem (32, 33). In addition, a large-scale meta-analysis of 23 studies involving 2,313 athletes showed that 56% of them were vitamin D deficient (34). Several studies have shown that the vitamin D levels of athletes vary by latitude (35), and athletes who engage in indoor sports have a greater incidence of vitamin D deficiency (32, 36). Moreover, the study by Aydin et al. (37) showed that the difference in vitamin D levels between outdoor and indoor athletes is evident, with 59% of outdoor athletes and 64% of indoor athletes generally experiencing vitamin D deficiency. These studies suggest that the level of vitamin D in athletes is related to the synthesis of vitamin D through sunlight exposure. In our study, through a mediation analysis, we found that sunlight exposure had a mediating effect on the relationship between PA and serum vitamin D levels and that the mediating effect of sunlight exposure in children aged 6–11 years and boys aged 12–19 years ranged from 28% to 46%, indicating that sunlight exposure plays an undeniable role in the relationship between PA and serum vitamin D levels.

What are the mechanisms related to PA and serum vitamin D levels? At present, the underlying mechanisms are unclear. PA can alter the balance of the body, change the levels of circulating media and hormones, and increase the energy demand of skeletal muscles and other important organs. Moreover, PA can promote bone and mineral metabolism, particularly calcium and phosphate metabolism (38), which are crucial for neuromuscular signaling, the biosynthesis of adenosine triphosphate (ATP), and other components of energy metabolism. Additionally, PA alters fat metabolism, which is a site at which inactive vitamin D is stored (39). In our study, we also found that BMI had a mediating effect on the relationship between PA and vitamin D levels. These changes caused by PA may lead to the acceleration of the release and activation of stored inactive vitamin D in the body, thereby increasing vitamin D levels to ensure calcium balance. Athletes may experience vitamin D deficiency due to continuous excessive exercise or insufficient levels of stored vitamin D. In our study, excluding the amount of time spent outdoors, BMI, age, etc., we did not find any statistically significant relationship between PA and serum vitamin D levels in children aged 3–5 years and girls aged 12–18 years, which may be due to insufficient muscle mass, insufficient PA volume or intensity, or insufficient vitamin D storage. In individuals with these conditions, changes in the calcium level cannot be stimulated by PA through muscle and bone metabolism, thereby preventing the activation and release of inactive vitamin D stored in fat. Further in-depth research is needed on the relationships and possible mechanisms through which PA promotes an increase in vitamin D levels in people of different ages.

A strength of our research is that we found that the relationship between PA and vitamin D levels varied among children and adolescents aged 3–19 years, providing a new perspective for further studies exploring the relationship between PA and vitamin D levels. Second, we found that the amount of time spent outdoors, which mainly means being exposed to sunlight, and BMI have mediating effects on the relationship between PA and vitamin D levels, which will contribute to studies of the relationship and mechanism between PA and vitamin D.

Our study extracted data from the Nutrition Examination Survey (NHANES), which is a cross-sectional survey of United States national health; therefore, the limitation of the present study was that the results cannot reveal a causal relationship between PA and serum vitamin D levels. However, the results of the present cross-sectional study can provide clues for further causal and mechanistic research. Second, the sample sizes of boys and girls aged 3–5 years were relatively small, 126 and 124, respectively, and the 95% confidence intervals of the groups aged 3–5 years were larger; therefore, the relationship between PA and vitamin D levels in children aged 3–5 years needs to be studied further after increasing the sample size.

## Data Availability

The datasets presented in this study can be found in online repositories. The names of the repository/repositories and accession number(s) can be found in the article/Supplementary material.
